# A randomized controlled trial of a smoking cessation smartphone application with a carbon monoxide checker

**DOI:** 10.1038/s41746-020-0243-5

**Published:** 2020-03-12

**Authors:** Katsunori Masaki, Hiroki Tateno, Akihiro Nomura, Tomoyasu Muto, Shin Suzuki, Kohta Satake, Eisuke Hida, Koichi Fukunaga

**Affiliations:** 1 Division of Pulmonary Medicine, Department of Medicine, Keio University School of Medicine, Tokyo, Japan; 2 Department of Internal Medicine, Saitama City Hospital, Saitama, Japan; 3 CureApp Institute, Karuizawa, Nagano, Japan; 4 Innovative Clinical Research Center, Kanazawa University (iCREK), Kanazawa, Ishikawa, Japan; 5 Department of Cardiovascular Medicine, Kanazawa University Graduate School of Medical Sciences, Kanazawa, Ishikawa, Japan; 6 CureApp, Inc, Tokyo, Japan; 7 Department of Biostatistics and Data Science, Osaka University Graduate School of Medicine, Osaka, Japan

**Keywords:** Preventive medicine, Respiratory tract diseases, Risk factors, Preventive medicine, Respiratory tract diseases

## Abstract

Evidence of the long-term efficacy of digital therapies for smoking cessation that include a smartphone application (app) is limited. In this multi-center randomized controlled trial, we tested the efficacy of a novel digital therapy for smoking cessation: the “CureApp Smoking Cessation (CASC)” system, including a CASC smartphone app, a web-based patient management PC software for primary physicians, and a mobile exhaled carbon monoxide (CO) checker. A total of 584 participants with nicotine dependence were recruited from October 2017 to January 2018, and allocated 1:1 to the CASC intervention group or the control group. Both groups received a standard smoking cessation treatment with pharmacotherapy and counseling for 12 weeks. Meanwhile, the intervention group used the CASC system, and the control group used a control-app without a mobile CO checker, each for 24 weeks. The primary outcome was the biochemically validated continuous abstinence rate (CAR) from weeks 9 to 24. The main secondary outcome was an extended CAR from weeks 9 to 52. Except for 12 participants who did not download or use the apps, 285 participants were assigned to the intervention group, and 287, to the control. CAR from weeks 9 to 24 in the intervention group was significantly higher than that in the control group (63.9% vs. 50.5%; odds ratio [OR], 1.73; 95% confidence interval [CI], 1.24 to 2.42; *P* = 0.001). The CAR from weeks 9 to 52 was also higher in the intervention group than that in the control group (52.3% vs. 41.5%; OR, 1.55; 95% CI, 1.11 to 2.16; *P* = 0.010). No specific adverse events caused by the CASC system were reported. Augmenting standard face-to-face counseling and pharmacotherapy with a novel smartphone app, the CASC system significantly improved long-term CARs compared to standard treatment and a minimally supportive control app.

## Introduction

Smoking is the leading cause of preventable death worldwide including Japan^1,2^. Approximately 20% of Japanese adults are current smokers (male: 29.4%, female: 7.2%, 2017 data)^3^, and smoking reportedly accounted for 129,000 deaths in 2007^4^ and 218,800 in 2014^5^. Therefore, reducing smoking prevalence is essential for preventing premature death.

Since 2006, patients with nicotine dependence can receive smoking cessation treatment covered by the Japanese national insurance system^6^. The program consists of five visits during a 12-week period, in which health care professionals provide counseling and pharmacotherapy, such as a nicotine patch or varenicline. However, the continuous abstinence rate (CAR) from weeks 9 to 12 is still as low as 50 to 60%^7–9^. In addition, long-term CARs have also been unsatisfactory; CARs from weeks 9 to 24 were around 40% at most, and from weeks 9 to 52, around 30% or less^7–9^. A better treatment tool is needed to improve smoking cessation outcomes and maintain abstinence.

Digital therapies, including a smartphone application (app), are promising tools for improving smoking cessation success. Several observational studies and pilot studies of apps for smoking cessation—including randomized controlled trials—have been conducted^10–15^. For example, Iacoviello et al. reported that the Clickotine smartphone app for smoking cessation achieved a 30% CAR at 8 weeks^15^. In addition, the feasibility of a mobile phone or an internet-based personal exhaled carbon monoxide (CO) measurement tool has been reported^14–20^. For example, Alessi and Wilson conducted studies to investigate the usability and feasibility of monitoring the concentration of exhaled CO by using mobile phones^19,20^. However, neither of the studies provided evidence that these strategies were more effective for long-term abstinence than standard smoking cessation support^14^. Thus, it would be a meaningful theme to test the additional effects of apps with a personal CO checker when utilized in conjunction with the standard treatment, including pharmacotherapy and face-to-face behavioral support.

**Fig. 1:**
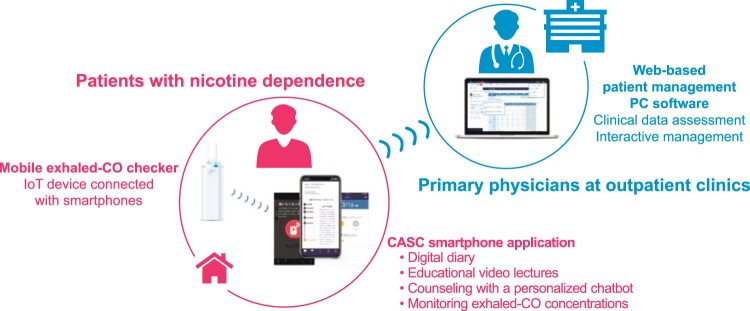
Overview of the CureApp Smoking Cessation system.

To address this gap, we have developed a novel digital therapy: the “CureApp Smoking Cessation” (CASC) system (Fig. 1). It consists of a CASC smartphone app for patients with nicotine dependence, a web-based patient management PC software for primary physicians, and a mobile exhaled CO checker. In a previous pilot study, we demonstrated that a prototype of the CASC smartphone app used together with a standard smoking cessation program yielded a higher CAR from weeks 9 to 24 compared with data from a Japanese national survey^7,8,21^.

In this study, we tested whether the addition of the CASC system to a standard smoking cessation program improved long-term CARs in patients with nicotine dependence.

## Results

### Recruitment and baseline characteristics

**Fig. 2:**
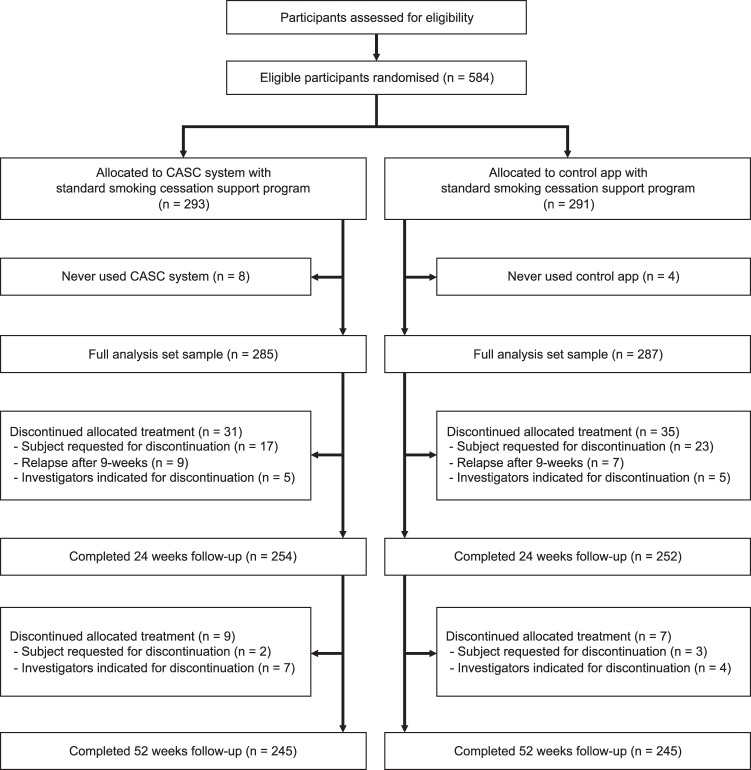
CONSORT flow diagram.

We randomized a total of 584 eligible participants from October 2017 to January 2018 (Fig. 2). Of all the participants, 12 (2.0%) did not download or never used either of the apps. Thus, 285 participants allocated to the CASC group and 287 participants assigned to the control group were included in the full analysis set. Completion rates at 24 weeks and 52 weeks were 89.1% (254/285) and 86.0% (245/285) in the CASC group, and 87.8% (252/287) and 85.4% (245 / 287) in the control group, respectively (Fig. 2).

Table 1 shows baseline characteristics of the trial participants. The participants’ mean age was 46 years (range: 23 to 80), and 75% were male. The median of the pack-years was 20 (range: 10 to 120). Varenicline was prescribed to 79% of the participants, and 20% received nicotine patches. There were no significant differences in baseline characteristics between the two groups.

**Table 1:** Baseline characteristics of the trial participants.

	Total (*N* = 572)	CASC (*N* = 285)	Control (*N* = 287)
Age	46 ± 11	47 ± 11	45 ± 12
Age ranges			
<40 years	171 (30)	75 (26)	96 (33)
40–49 years	179 (31)	97 (34)	82 (29)
50–59 years	159 (28)	82 (29)	77 (27)
≥60 years	63 (11)	31 (11)	32 (11)
Male sex	426 (75)	216 (76)	210 (73)
Smoking history			
Years of smoking	25 (18–33)	25 (19–32)	24 (17–33)
Cigarettes per day	20 (15–20)	20 (15–20)	20 (15–20)
Pack-years	20 (14–30)	21 (14–30)	20 (13–31)
Exhaled CO (ppm)	17 ± 11	17 ± 10	18 ± 11
TDS score	7.7 ± 1.5	7.7 ± 1.4	7.8 ± 1.5
FTND	5.3 ± 2.1	5.2 ± 2.0	5.3 ± 2.1
Comorbidities			
Cardiovascular diseases	93 (16)	45 (16)	48 (17)
Respiratory diseases	93 (16)	48 (17)	45 (16)
Psychiatric diseases	31 (5)	12 (4)	19 (7)
Prescribed Medication			
Varenicline	454 (79)	227 (80)	227 (79)
Nicotine patch	114 (20)	56 (20)	58 (20)
No medication	4 (1)	2 (1)	2 (1)

Data include mean ± standard deviation, number (%), or median (interquartile range) scores.

*CO* carbon monoxide, *FTND* Fagerström test for nicotine dependence, and *TDS* tobacco dependence screener.

### Outcomes

**Fig. 3:**
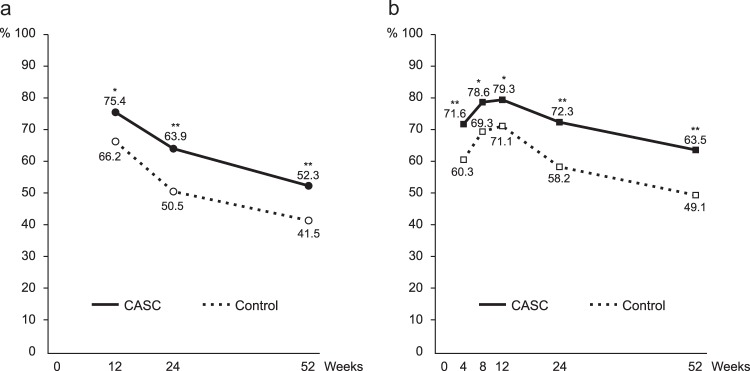
Biochemically validated smoking abstinence rates. **a** Continuous abstinence rates from weeks 9 to 12, 9 to 24, and 9 to 52. **b** Seven-day point prevalence abstinence at weeks 4, 8, 12, 24, and 52. *P < 0.05, **P < 0.01.

Regarding the primary outcome, biochemically validated CARs from weeks 9 to 24 were significantly higher in the CASC group (63.9%; 182/285) than the control (50.5%; 145/287) (odds ratio [OR], 1.73; 95% confidence interval [CI], 1.24 to 2.42, *P* = 0.001) (Fig. 3).

In the secondary outcomes, CARs from weeks 9 to 52 were also significantly higher in the CASC group than the control (52.3%; 149/285 vs. 41.5%; 119/287; OR 1.55; 95% CI, 1.11 to 2.16; *P* = 0.010). In other secondary outcomes, CARs from weeks 9 to 12 were higher in the CASC group than the control (75.4%; 215/285 vs. 66.2%; 190/287; OR, 1.57; 95% CI, 1.09 to 2.27; *P* = 0.016). Regarding the 7-day point prevalence abstinence (PPA), ORs were 1.67 at week 4 (95% CI, 1.17 to 2.38; *P* = 0.004), 1.63 at week 8 (95% CI, 1.11 to 2.39; *P* = 0.012), 1.56 at week 12 (95% CI, 1.06 to 2.30; *P* = 0.024), 1.88 at week 24 (95% CI, 1.33 to 2.68; *P* < 0.001), and 1.80 at week 52 (95% CI, 1.29 to 2.52; *P* < 0.001) (Fig. 3). The time to the first lapse after the quit date was longer in the CASC group (median, 267 days; interquartile range [IQR], 5 to 340) than in the control group (median, 19 days; IQR, 4 to 330) (*P* < 0.001). The Mood and Physical Symptoms Scale (MPSS: urge total and total excluding urges), the 12-item French version of the Tobacco Craving Questionnaire (FTCQ-12: emotionality, expectancy, purposefulness, and general craving score), and the Kano Test for Social Nicotine Dependence (KTSND) scores decreased from the baseline more prominently in the CASC group compared to the control (*P* < 0.05) (Table 2).

**Table 2:** Changes in MPSS, FTCQ-12, and KTSND scores from baseline to weeks 24 and 52, adjusted by covariates.

	CASC (*N* = 285)	Control (*N* = 287)	*P-* value^a^
Change from weeks 0 to 24			
MPSS urge total	−1.87 [−2.01 to −1.72]	−1.73 [−1.88 to −1.59]	0.009
MPSS total excluding urges	−0.59 [−0.72 to −0.47]	−0.42 [−0.56 to −0.29]	<0.001
FTCQ-12 emotionality	−1.70 [−1.89 to −1.51]	−1.32 [−1.49 to −1.14]	<0.001
FTCQ-12 expectancy	−2.46 [−2.68 to −2.25]	−2.16 [−2.37 to −1.95]	0.002
FTCQ-12 compulsivity	−1.74 [−1.94 to −1.54]	−1.74 [−1.93 to −1.55]	0.202
FTCQ-12 purposefulness	−2.84 [−3.10 to −2.58]	−2.17 [−2.44 to −1.90]	<0.001
FTCQ-12 general craving score	−2.09 [−2.25 to −1.93]	−1.78 [−1.93 to −1.63]	<0.001
KTSND	−7.0 [−7.7 to −6.2]	−3.9 [−4.5 to −3.2]	<0.001
Change from weeks 0 to 52			
MPSS urge total	−1.82 [−1.98 to −1.67]	−1.65 [−1.81 to −1.49]	0.007
MPSS total excluding urges	−0.52 [−0.64 to −0.39]	−0.42 [−0.54 to −0.29]	0.061
FTCQ-12 emotionality	−1.60 [−1.80 to −1.41]	−1.21 [−1.39 to −1.03]	0.001
FTCQ-12 expectancy	−2.39 [−2.60 to −2.19]	−2·10 [−2.33 to −1.88]	0.002
FTCQ-12 compulsivity	−1.71 [−1.90 to −1.52]	−1.55 [−1.75 to −1.35]	0.019
FTCQ-12 purposefulness	−2.84 [−3.10 to −2.58]	−2.00 [−2.28 to −1.73]	<0.001
FTCQ-12 general craving score	−2.03 [−2.19 to −1.87]	−1.65 [−1.81 to −1.48]	<0.001
KTSND	−5.9 [−6.6 to −5.3]	−4.1 [−4.8 to −3.5]	<0.001

Mean [95% CI] scores are provided. Covariates: medications (varenicline, nicotine patch, or none) and medical institutions.

*MPSS* Mood and Physical Symptoms Scale (ranging from 0 to 5), *FTCQ-12* French version of the Tobacco Craving Questionnaire 12 (ranging from 1 to 7), *KTSND* Kano Test for Social Nicotine Dependence (ranging from 0 to 30), *CI* confidence interval.

^a^Analysis was based on analysis of covariance.

### Adverse events

In all, 67.4% (192/285) of the CASC group and 63.8% (183/287) of the control had treatment-emergent adverse events, including viral upper respiratory tract infections (20.4% in the intervention group vs. 12.9% in the control), nausea (18.6% vs. 22.3%), influenza (6.0% vs. 3.5%), headache (5.3% vs. 4.2%), constipation (4.9% vs. 5.6%), insomnia (4.6% vs. 2.8%), weight gain (3.9% vs. 2.4%), somnolence (3.5% vs. 5.9%), abdominal discomfort (3.2% vs. 2.1%), and/or contact dermatitis (2.8% vs. 3.1%). No specific adverse events related to the CASC system were reported, and some events were related to either nicotine withdrawal symptoms or side-effects of smoking cessation medications.

### App usage and acceptability

In the CASC group, the ratios of participants using the smartphone app and continuing the study were 97.5% (270/277) at week 2, 100% (276/276) at week 4, 99.3% (271/273) at week 8, 99.6% (259/260) at week 12, and 99.6% (252/253) at week 24. Further, the median compliance rate of writing in the digital diary was 71.4% (IQR, 44.3 to 96.4%). Of 29 animated videos and educational tutorials provided via the CASC smartphone app, the median number of completed viewings followed by self-reported comprehension and learning was 18.5 (IQR, 8 to 24).

The median ratios of daily digital diary entries of the participants were 88.0% among 182 successful quitters from weeks 9 to 24, and 45.6% of 103 participants who failed to quit. The median number of the educational tutorials that successful quitters reported they understood well was 21, whereas the median number that failed participants reported they understood well was 11. In addition, successful participants reported they practiced app-recommended behavior to avoid smoking 136 times (median) cumulatively from weeks 9 to 24, while the failed participants practiced 80 times (median).

There was no report suggesting a discrepancy between daily CO levels at home and self-reported quitting status.

## Discussion

This was the first large randomized controlled trial to evaluate the efficacy of a novel digital therapy for smoking cessation, the CASC combination system of a smartphone app, patient management PC software for physicians, and a mobile CO checker. We found that the CASC system significantly improved long-term CARs in patients with nicotine dependence, when used with standard smoking cessation treatments including pharmacotherapy.

This trial has several important findings. First, the primary outcome reached statistical significance: biochemically verified CARs from weeks 9 to 24 were significantly higher in the CASC group (63.9%) than in the control (50.5%). The impact of the additional 13.4% improvement was comparable to and even better than the effect of varenicline when compared with placebos in previous reports^9,22^. In intention-to-treat analysis conducted as post-hoc data analysis, including all randomized participants, consistent significance and effect size were also demonstrated (data not shown). The difference of the CARs between the two groups cannot be explained by insufficient treatment of the control group, because the CARs for the control group were still higher than those cited by a previous report of Japanese smokers treated with varenicline^9^. Generally, the surveys and studies conducted in Japan, including this trial, yielded relatively high quit rates compared to those conducted in other countries^7–9,14,22–24^. Although the exact reason for this is unclear, it could be attributed to some unique features of the national smoking cessation program in Japan: intensive face-to-face counseling provided by both physicians and nurses; high prescription rates of varenicline (>70%); to exclude the patients who do not intend to quit smoking immediately; and to obtain written agreement to follow the smoking cessation program from each patient at the initial visit^6–8^. Taken together, this study indicates that digital therapeutics with the CASC system can be a useful supplementary treatment method for nicotine-dependent patients who are treated with face-to-face counseling and pharmacotherapy in Japan.

Second, the effects of the CASC system continued up to 52 weeks, even after the participants uninstalled the CASC smartphone app at week 24. While in previous randomized trials, CARs to week 52 using varenicline and/or nicotine patches were only 20–35%^9,22–24^, the present study yielded a CAR of over 50% from weeks 9 to 52. This long-term effect might stem from CASC smartphone app-provided tutorials and counseling, along with self-management practices to cope with withdrawal symptoms and cravings for smoking. Indeed, the MPSS and FTCQ-12 scores improved more in the intervention group than in the control throughout the study, indicating that the CASC users may not have struggled as much against cessation-related withdrawal symptoms and cravings. In addition, the improvement of the KTSND scores indicated that CASC may mitigate the severity of social and psychological dependence on smoking and contribute to preventing relapse.

Third, daily use of app functions by successful quitters, such as a digital diary, video tutorials, and behavioral therapy provided by the chatbot was more frequent than among failed participants in the CASC group. Although this finding is not conclusive, these app functions seemed to be important for the maintenance of high adherence rates. Some studies have reported that adherence rates for using a smartphone app for cessation were associated with an increased likelihood of abstinence^15,25,26^. Thus, the usability of the CASC app may contribute to the high success rate and the prevention of relapse. The web-based PC software for physicians also assisted them in following participants’ progress via a cloud system and encouraging participants to continue to use the app during the study period. In addition, the novelty and usability of the system could attract the participants enough to retain high engagement rates via the unique functions of the system, such as a mobile exhaled CO checker at home, friendly animations for smoking cessation education, and bidirectional counseling with a chatbot. For example, measuring the level of exhaled CO once a day and recording it into a digital diary may provide an effective goal-oriented form of encouragement for users to maintain quitting.

The strengths of this study are as follows: (1) it was the first large randomized controlled trial to evaluate the efficacy of a novel smartphone system for smoking cessation integrated with a mobile exhaled CO checker to supplement standard treatment with face-to-face behavioral support and pharmacotherapy; (2) it included a long-term observation period (24 and 52 weeks); (3) it measured biochemically validated CARs; and (4) it measured the additional clinical and psychological efficacy to counseling and pharmacotherapy when supplementing a standard smoking cessation program. The study also had some limitations: (1) participating institutions were mostly restricted to urban areas in Japan, where smartphone usage rates were high; (2) a majority of the participants were middle-aged, and the standard deviations for age were relatively small; (3) a lack of socio-educational data of participants, which may affect the results of smoking cessation; (4) participants were limited to those with technological literacy in using mobile devices; (5) the primary endpoint (continuous abstinence from weeks 9 to 24) did not align with the Russell standard of recording 6-month continuous abstinence in smoking cessation trials^27^; (6) compensation of 90 US dollars per visit may have impacted both app engagement and quitting success, although compensation was provided to both groups; (7) medical staff at clinics were not blinded to group allocation (single blinded study); and (8) it is notable that there was no discrepancy between exhaled CO at home and self-reported quit status, suggesting that participants were motivated to respond honestly, although this extremely high consistency may not be generalizable to other healthcare settings. In addition, there is a possibility that the control group may have guessed that they were provided with the control app because of its minimal functions, which could have discouraged them from better adherence, even though the trial staff never mentioned group assignment to the participants.

In conclusion, a novel digital therapy, the CASC system, significantly improved a long-term CAR from weeks 9 to 24 in conjunction with standard smoking cessation treatment in patients with nicotine dependence. Digital therapy for smoking cessation may be a promising strategy to reduce smoking prevalence worldwide, and future research on a global scale is warranted.

## Methods

### Study design

This was a multi-center, randomized, controlled, open-label trial (Fig. 2). A detailed study protocol was presented elsewhere^28^. Briefly, participants were recruited from 31 smoking cessation clinics in Japan, from October 2017 to January 2018, and allocated 1:1 to the CASC intervention group and the control group. The intervention group used the CASC system, and the control group used the control app, each for 24 weeks, in addition to a 12-week standard smoking cessation treatment. In reference to the varenicline maintenance therapy for 24 weeks^22^, we were interested in whether or not the effectiveness of the CASC system could be maintained after discontinuation of using the app, and we limited access to the app to 24 weeks and evaluated CARs up to 52 weeks. The study was performed in compliance with CONSORT statements. The protocol and written informed consent forms were reviewed and approved by the Institutional Review Board at Keio University School of Medicine and all affiliated institutions. This trial was registered at the University Hospital Medical Information Network (UMIN) Clinical Trials Registry (UMIN000031589).

### Participants

We recruited nicotine-dependent adults who visited outpatient clinics to receive smoking cessation treatment under the Japanese National Health Insurance program. Physicians at each clinic screened the patients and obtained written informed consent. The baseline data were collected using a self-administered questionnaire at the initial visit. The program consisted of five visits during a 12-week period, and counseling and pharmacotherapy, including nicotine patch or varenicline were provided by physicians^6^. Inclusion criteria were as follows: adult current smokers who (1) were diagnosed with nicotine dependence (tobacco Dependence Screener [TDS] score ≥5 points)^29^; (2) had a smoking history of pack-years ≥10; (3) intended to quit smoking immediately; (4) agreed to participate in a smoking cessation treatment program with written informed consent; and (5) could use a smartphone without difficulty (OS: Android® 5.0 and above, or iOS® 8.0 and above). “Difficulty of using smartphone” was defined based on participants’ self-reports; that is, participants who used smartphones in their daily lives were included in the trial unless they declined participation due to difficulty in using smartphones. Individuals who met any of the following criteria were excluded from the study: (1) had severe mental illness; (2) could not come to the follow-up clinic visits during the trial period; (3) started taking a smoking cessation medication within 1 year before the registration; or (4) planned to use any smoking cessation aids and/or to participate in any kind of smoking cessation activities (not limited to smoking cessation therapy) outside of the trial. During the trial period, physicians could report discontinuation from the study when the participants (1) were lost during follow-up and could not be contacted by the investigators; (2) reported incompatibility between the study app and their smartphone (e.g., inability to install the app); or (3) no longer intended to quit smoking.

### Randomization and masking

Randomization was performed for eligible participants by a computer-generated random sequence with stratification for smoking cessation medications and study sites. The passcodes to download the apps were provided to all participants; a mobile CO checker was provided only to the intervention group, meaning that the study staff was unavoidably aware of the group assignment. Participants were not informed before assignment that the study system included not only apps but also a mobile CO checker, meaning that the participants were unaware of the group to which they were allocated. Thus, this was a single blind study.

### Intervention and control smartphone apps

The CASC system consisted of the smartphone app, a connected cloud system to upload data, a paired mobile exhaled CO checker device, and a web-based PC software for physicians (Fig. 1). The physician provided a prescription code to each study participant during the initial visit. Participants downloaded the app on their own smartphones, activated it with the passcode, and began to use the app on the day they started taking medications. The baseline data were also collected from the app, as entered by the participants. These data were securely sent to cloud storage, and the system assembled a tailor-made smoking cessation program that was best suited for each participant, based on their initial information. The personal data stored on the cloud system could be reviewed only by their primary physicians.

The CASC smartphone app had four distinct components to maximize the therapeutic effects of pharmacotherapy and behavioral therapy (Supplementary Movie 1 and Supplementary Table 1): (1) keeping a smoking cessation digital diary with daily entries; (2) viewing animated videos and educational tutorials designed to help users learn how to quit smoking (Supplementary Table 2); (3) participating in interactive counseling with a personalized chatbot (an automated guidance system for communication with users several times a day); and (4) measuring and recording exhaled CO concentrations at home once a day. A web-based PC software version of CASC enabled physicians to review participants’ progress at the clinic, and also provided useful tips to assist the physicians in counseling participants effectively, following the national clinical guidelines^6^.

The control-group app provided only the basic functions of the CASC app: (1) showing the user guide (how to use the app); (2) entering participants’ profiles and setting a target quit date; (3) displaying the schedule of five visits during the 12-week treatment with a summary of objectives of each visit; (4) showing the date of the next appointment; (5) getting the contact form for technical support; and (6) displaying an app version, privacy policy, and administrative information. The control app was not accompanied by a mobile CO checker.

Exhaled CO concentrations were measured by the medical staff at each clinic visit (0, 2, 4, 8, 12, 24, and 52 weeks) for all participants in both groups and were used for analysis. Both apps were uninstalled at the week 24 clinic visit. Compensation of 10,000 yen (i.e., 90 US dollars) was provided to participants at each clinic visit, including travel expenses.

### Primary and secondary outcomes

The primary outcome was a biochemically validated CAR from weeks 9 to 24. CAR was computed from week 9 according to the Japanese national survey by the Ministry of Health, Labor and Welfare and the Phase 3 clinical trial of varenicline in Japan^7–9^. The success of smoking cessation was defined as (1) the self-reporting of continuous abstinence and (2) an exhaled CO concentration ≤10 ppm, measured by medical staff at each clinic visit. The secondary outcomes were as follows:

- biochemically validated CAR from weeks 9 to 12 and 9 to 52;
- The 7-day PPA at weeks 4, 8, 12, 24, and 52;
- Changes in scores on the following three scales related to smoking cessation: MPSS^30^, an assessment of withdrawal symptoms; FTCQ-12^31^, a scale for assessing cravings; KTSND^32^, an evaluation of misperceptions about smoking;
- Time to first lapse after the quit date;
- The usage rate of either the CASC system or the control app; and
- The presence of product problems or adverse events.

### Scales and scores

In this study, we used several kinds of score systems that reflected the severity of nicotine dependence. For all score systems, higher scores are related to worse outcomes. The content of each score in detail is as follows:

- TDS [minimum: 0, maximum: 10] is a self-administered 10-item scale, and is based on the Diagnostic and Statistical Manual, fourth edition (DSM-IV) and ICD-10 definitions of dependence^29,33^. It is a required diagnostic tool for nicotine dependence in Japan; scores of 5 and greater are diagnostic indicators and a screening criterion for the coverage of nationally insured treatment under the National Health Insurance Program.
- Fagerström Test for Nicotine Dependence (FTND) [minimum: 0, maximum: 10] is widely used for assessing physical nicotine dependence to predict difficulty of abstinence.
- MPSS [minimum: 1, maximum 5] was developed to assess cigarette withdrawal symptoms by evaluating urges and cravings for smoking. It is composed of the factors of urges for smoking (in terms of frequency and strength), depressed mood, irritability, restlessness, hunger, and poor concentration. All items are rated on a 5-point Likert scale: “not at all” (1), “slightly” (2), “somewhat” (3), “very” (4), and “extremely” (5).
- FTCQ-12 [minimum: 1, maximum 7] is a brief, valid, and reliable self-report of tobacco craving. Items are rated on a Likert-type scale from 1 (strongly disagree) to 7 (strongly agree). Factor scale scores for each participant were obtained by summing the items in each factor (i.e., emotionality, expectancy, compulsivity, and purposefulness) and dividing by the number of items, yielding a composite score ranging from 1 to 7.
- KTSND [minimum: 0, maximum: 30] consists of ten questions to evaluate the psychological and social aspects of smoking dependence and reflects the stages for smoking cessation (e.g., “Does smoking sometimes enrich people’s lives?”). Items are rated on a Likert-type scale: “definitely no” (0), “probably no” (1), “probably yes” (2), and “definitely yes” (3). Final scores are obtained by summing each score for the ten questions.

### Statistical analysis

All statistical analyses were performed with the full analysis set based on the intention-to-treat principle. We expected that CASC intervention for 24 weeks could obtain an additional effect of 12% on the CAR compared to the control group, in reference to a report of maintenance therapy with varenicline for 24 weeks that obtained an additional effect of 12% on the CAR compared with a placebo treatment^22^. A sample size of 290 for each group was calculated, which would have 80% power with a two-tailed significance level of 0.05. For the CAR and PPA at each defined period, a logistic regression model was used to calculate the OR and the 95% CI, adjusted for prescribed smoking cessation medications (i.e., varenicline, nicotine patch, or none). Analyses of the MPSS, FTCQ-12, and KTSND scale data were conducted with analysis of covariance, adjusted for the study sites and medications. A significance level of 0.05 was adopted in a two-tailed test, and dropout participants were considered to have failed. We used SAS statistical software, version 9.4 or higher, (SAS Institute Inc., NC, USA) for all analyses.

### Reporting summary

Further information on research design is available in the Nature Research Reporting Summary.

## Supplementary information

Supplementary Movie 1

Supplementary Information File

Reporting Summary

## Data Availability

An anonymized dataset from this trial is available from the corresponding author (hrk12tateno@me.com) upon publication. The investigator may only use the data for the purpose outlined in the request, and redistribution of the data is prohibited.
