# Supplementary material for: A randomized controlled trial of a smoking cessation smartphone application with a carbon monoxide checker
Source: NPJ Digit Med. 2020 Mar 12;3:35. doi: 10.1038/s41746-020-0243-5 (PMC7067789; doi:10.1038/s41746-020-0243-5)
Supplement: Supplementary file 2 — Supplementary Information File [file 41746_2020_243_MOESM2_ESM.pdf]

Supplementary Information File

A randomized controlled trial of a smoking cessation smartphone application with a carbon monoxide checker

Katsunori Masaki, Hiroki Tateno, Akihiro Nomura, Tomoyasu Muto, Shin Suzuki, Kohta Satake, Eisuke Hida, Koichi Fukunaga

Supplementary Table 1. The functions of the CureApp Smoking Cessation system compared with the control application

Components / Functions	CASC	Control
Digital dialy<br>• Recording whether taking medicines on the day<br>• Recording body weight daily<br>• Recording how much urge to smoke experienced<br>• Recording behavioural therapies practiced on the day	○	-
Educational video tutorials in animation (cf. Table E2)<br>• Learning what nicotine dependence and withdrawal symptoms mean<br>• Relapse prevention training<br>• Users could review the tutorials repeatedly as needed	○	-
Interactive counselling with a personalized chatbot<br>• Automatically starting chatting several times a day<br>• Boosting motivation and providing behavioural therapy<br>• Users also could bring up the chatbot whenever they needed advices to manage urge to smoke	○	-
Daily measurement of exhaled-CO<br>(automatically transported to smartphone by Bluetooth)	○	-
Guidance for physicians<br>• Reviewing patients' progress<br>• Tips for counselling patients	○	-
Other basic functions<br>• Entering personal information and setting the quit date<br>• Guidance of next outpatient clinic appointment<br>• Getting the contact form for technical support<br>• Displaying app version, privacy policy, and administrative information	○	○

Supplementary Table 2. Schedule of animated videos and educational tutorials by CureApp Smoking Cessation smartphone application.

No.	Lecture title
1	Behavioral contract
2	Nicotine dependence is a disease
3	Nicotine dependence has two components<br>One: physical dependence
4	Nicotine dependence has two components<br>Two: psychological dependence
5	Build a smoke-free environment
6	Change your behavior patterns
7	Find something else rather than smoking
8	Declaration of smoking cessation
9	Withdrawal symptoms
10	Smoking cessation techniques: check-ups
11	Keep making an effort for smoking cessation
12	Self-assertiveness workshop
13	Praise yourself when you succeed
14	Visit the clinic more often, and your will gain higher success
15	Body weight may increase temporally, while smoking cessation is much better for your health
16	Positive impact of smoking cessation on beauty
17	Build a perception that you may fail to stop smoking
18	Decline the call for smoking together by friends
19	It’s not because you are weak-willed that you cannot quit smoking
20	Smoking even one piece of cigarette resets all your efforts for smoking cessation
21	You cannot relieve your stress through smoking
22	Relapse prevention training
23	Control your smoking mind
24	You reach the middle of the program
25	Graduation (deployed at the completion of the 5th outpatient-clinic visit)
26–28	Prevention program for relapse of smoking

30	Final chapter

Supplementary Movie 1.

A representative video tutorial of CureApp Smorking Cessation.
